# Barriers and facilitators to antiretroviral adherence and retention in HIV care among people living with HIV in the Comarca Ngäbe-Buglé, Panama

**DOI:** 10.1371/journal.pone.0270044

**Published:** 2022-06-16

**Authors:** Amanda Gabster, Eliana Socha, Juan Miguel Pascale, Gonzalo Cabezas Talavero, Alezander Castrellón, Yaremis Quiel, César Gantes, Philippe Mayaud

**Affiliations:** 1 Instituto Conmemorativo Gorgas de Estudios de la Salud, Panamá, Panamá; 2 London School of Hygiene and Tropical Medicine, London, United Kingdom; 3 Sistema Nacional de Investigación, Secretaría Nacional de Ciencia, Tecnología e Innovación, Panamá, Panamá; 4 Community Development Network of the Americas, Panamá, Panamá; 5 Facultad de Medicina, Universidad de Panamá, Panamá, Panamá; 6 Ministerio de Salud, Comarca Ngäbe-Buglé, Panamá, Panamá; Kasturba Medical College Manipal, INDIA

## Abstract

**Introduction:**

Panama’s HIV epidemic is far from under control. One of the populations with the fastest-growing epidemic among the Indigenous peoples of the Comarca Ngäbe-Buglé (CNB). The CNB is an administratively autonomous Indigenous region in Western Panama that is home to over 200,000 individuals of Ngäbe and Buglé ethnicities. This population is unique and, in several ways, represents the early stages of the AIDS epidemics in high-income countries. The CNB is the most impoverished region in Panama and is relatively isolated from outside influences, with limited roads, electricity, and an internet connection, including medical assistance. Around 1.5% of all rapid HIV tests are positive, compared to a national prevalence of 0.9%; in CNB, diagnosis tends to be late. In CNB, 56.3% of individuals had an initial CD4 count of <350 cells/mm3. Antiretroviral treatment (ART) dropout in this region is five times higher than the national average; there is high early mortality due to opportunistic infections. Using the Social-Ecological Theory for Health as a framework, this study aims to describe the facilitators and barriers associated with ART adherence and retention in HIV care among people living with HIV (PLHIV) in the CNB. A better understanding of factors that obstruct adherence could lead to more effective HIV care and prevention in CNB.

**Methods:**

We conducted 21 semi-structured interviews with PLHIV who reside across all three regions of the CNB and have attended an antiretroviral (ART) clinic at least once. Deductive thematic analysis was used to uncover themes related ART adherence and retention in HIV care at the individual, social and structural levels.

**Discussion:**

This unique, isolated population of rural Indigenous peoples has high infection rates, late diagnosis, poor ART adherence, and high AIDS-related death rates. The CNB is an important region to examen ART adherence and retention in care. We determined that psychological health, social support, and discrimination acted as individual-level facilitators and barriers to adherence and retention. Notably, structural barriers included difficult access to ART care due to travel costs, ART shortages, and uncooperative Western/Traditional medical systems. Recommended interventions used in other Low- and Middle-Income settings include increasing peer and family-level support and community knowledge and understanding of HIV infection. Additionally, our study suggests structural interventions, including decreasing the cost and distance of traveling to the ART clinic, by decentralizing services, decreasing food scarcity, and increasing collaboration between Western and Traditional providers.

## Introduction

The Comarca Ngäbe-Buglé (CNB) is a politically semi-autonomous region in Western Panama that is home to over 200,000 people of Ngäbe and Buglé ethnicities, making up 5.4% of the total Panamanian population [1]. The majority of adults of the Comarca speak Ngäbere or Murire as a first language at home, while Spanish is used widely in community settings and as the only language in public institutions such as schools and health centers [2, 3]. The CNB has the highest levels of poverty (defined as <$1.90/day) and extreme poverty (<$1.25/day) in Panama: in 2015, it was estimated that between 56 and 78% of households across the CNB lived in extreme poverty [4]. In addition, the CNB is relatively isolated from the rest of Panama, with limited roads, electricity, and internet access [5].

In 2018, the HIV prevalence in Panama among 15-49-year-old individuals was 0.9%, whilst in the CNB, 1.5% of tests performed were positive [6, 7]. Since 2000, access to antiretroviral therapy (ART) has been provided for free to all HIV-positive individuals in Panama [8]. However, Panamá currently has an uncontrolled epidemic, and the CNB has seen a spike in new infections while AIDS-related deaths continue to rise [9]. Ministerio de Salud, 2019. Recolección de indicadores nacionales e internacionales, Unpublished). Retention in HIV care is the regular participation in HIV care, while adherence is the use of ART regularly as a health professional indicates. Together, high retention and adherence should lead to high HIV viral load suppression rates, reduction of ART resistance, decrease in ongoing HIV transmission [10], and overall survival [11, 12]. Unfortunately, in the CNB, access to ART and HIV-related care is only available in 2 of 14 health centers, which, compounded by lack of trust in the Western medical system, has resulted in poor community testing, low ART initiation and adherence, and low community viral suppression rates.

This lack of access to treatment, low ART adherence and retention in HIV care, are reflected in epidemiological reports. For example, whilst HIV was the ninth most common cause of death among men and women nationwide in 2018, it was the first cause of death among men and second among women in the CNB (Ministerio de Salud, 2018, National HIV database, Unpublished). In 2019, late diagnosis for HIV was common, where 56% of all patients had an initial CD4 count at <350 cells/mm^3^ and 25% at <200 cells/mm^3^. ART adherence has been less than optimal in the CNB. In 2019, 30% of patients in CNB abandoned ART and HIV care—the majority within the first year after their diagnosis—compared to the national average of 6.5% (Ministerio de Salud, 2019, Recolección de indicadores nacionales e internacionales, Unpublished). Consequently, viral suppression (<1000 copies/ml), was reported to be only 52% among men and 49.0% among women in the CNB in 2019.

Considering the high mortality due to AIDS-related illnesses in the CNB, we undertook a qualitative study among HIV-positive individuals to describe the individual, social, structural levels of facilitators and barriers to ART adherence and retention in HIV care in the CNB, as better comprehension of these factors will help in the development of more effective, culturally congruent HIV care for people living with HIV (PLHIV) in the CNB. The Social-Ecological Theory for Health has been used previously to describe factors associated with ART adherence and retention in care [13, 14]. Three primary levels have been associated with the model: individual, social, and structural levels [15, 16]. Individual-level factors include personal attitudes and beliefs; the social-level factors include interpersonal interaction with peers, friends, family, and peers; the structural-level factors include the healthcare system, poverty community organizations, and policy.

## Methods

### Study design and participants

The study design and topics of discussion arose during a previous research study on HIV and other sexually transmitted infections (STI) conducted among young people in the CNB [17]. It became evident that participants who had tested positive for HIV during this study had difficulty maintaining adherence and retention in care. In order to understand what factors may influence this difficulty, we conducted interviews with PLHIV at the two existing ART clinics in CNB or at a private location of their choice in the interviewee’s community. Local clinicians who provide ART to the community (authors CG and YQ) selected participants from their clinics using purposive sampling from their patients’ files to find those with varying levels of adherence and retention success, where some were previously non-adherent individuals who were adherent at the time of the study, those who had intermittent adherence over time, and those who always maintained high adherence. The interview schedule **(Table 1)** was piloted among ten non-PLHIV in two CNB communities, for language understanding and acceptability before undertaking the study.

**Table 1:** Interview schedule.

Key questions and themes	Prompts and sub-questions
Can you tell me a bit about your diagnosis? What happened after?	• What was it that motivated you to get treatment? • How were you treated in the health center or hospital?
Tell me about the treatment you are taking now.	• Why did you select this treatment?Or why did you stop taking treatment or go to the ART clinic? • How do you feel about taking this treatment? • Have you taken traditional medicine? Can you tell me about that?
Tell me a bit about the difficulties you have faced while trying to access ART.	• How much does it cost for you to get your ART? • Have you ever had a bad experience in the system? Did this affect your ART use?
What has been the process of adaptation to your diagnosis	•How do you feel about your diagnosis? • What plans do you have for your future?
How has your diagnosis changed or affected your relationship with your family or friends?	• What have been the reactions of your family and friends? • How do they support you? • How do they treat you differently?
And the rest of your community?	• Have you felt you were discriminated against? Can you tell me about it? • What are the opinions of your community regarding HIV, and how do these opinions affect you?
What are some things that have affected your wellbeing since you were diagnoses?	• Is there something that has affected you negatively? What happened, and how did it affect you? • Tell me about your spiritual beliefs and how these affect your diagnosis.
Tell me what you have learned about HIV since you were diagnosed.	• What would you like to learn more about? What can the clinic do better? • What kind of orientation do you wish your family received?

Authors AG, ES, and AM conducted the semi-structured interviews in Spanish. Interviews took 20–40 minutes and were conducted until saturation of key themes regarding ART adherence and retention in care. A digital recorder was used to record the interviews. Interview themes and questions are found in **Table 2**.

**Table 2:** Participant characteristics, Comarca Ngäbe-Buglé, Panama, 2018.

Pseudonym	Gender	Age range	Region of CNB
*Chäti*	Man	31–50	Southern
*Icho*	Man	18–30	Southern
*Chilo*	Man	18–30	Southern
*Pancho*	Man	18–30	Northern
*Chiko*	Man	31–50	Northern
*Chöli*	Man	18–30	Northern
*Itoli*	Man	31–50	Southern
*Jichi*	Man	18–30	Southern
*Cheko*	Man	31–50	Southern
*Mirono*	Man	18–30	Southern
*Jebi*	Woman	31–50	Northern
*Cheyei*	Man	18–30	Southern
*Chichiko*	Man	18–30	Northern
*Mechi*	Woman	31–50	Southern
*Joti*	Man	18–30	Northern
*Nichi*	Man	18–30	Southern
*Jichi*	Man	18–30	Southern
*Choi*	Man	18–30	Northern
*Metiko*	Woman	18–30	Northern
*Belisi*	Woman	31–50	Southern
*Kötä*	Man	31–50	Northern

### Analysis

The interviews were transcribed by authors GCT and AC and then translated from Spanish to English by AG. A research assistant cross-checked the translation. Deductive thematic analysis uncovered themes of individual, social and structural factors related to ART adherence. Interview transcripts were first read, then data were broken into codes using NVIVO12 software (QSR International, Melbourne, Australia). Codes were organized into themes that were part of the original codebook; other themes were allowed to emerge that were not directly classifiable as individual, social and structural factors, however, they lay within the interaction between these factors. AG coded interviews: four interviews were checked for interrater reliability with author ES. Contradictions were agreed upon. Saturation of themes and codes was reached within 12 interviews; coding was completed for all interviews.

### Ethical approval

The research was approved by the Comité Nacional de Bioética de la Investigación de Panamá (EC-CNBI-20180836). All participants were ≥18 years and gave written informed consent prior to initiating the interviews.

## Results

Between April and December 2019, we conducted 21 semi-structured interviews with PLHIV, 12 from the Southern mountainous regions of Nedrini and Kädriri, and nine from the Northern region of Ñö Kribo. All participants were of Ngäbe or Buglé ethnicities, 81% were male (**Table 2).** Of participants, 62% (n = 13) had missed at least two clinic visits for ART collection or CD4/viral load testing within the two years prior to undertaking the study.

We present our findings following the Social-Ecological model, in terms of facilitators and barriers to ART adherence and retention in care at the individual, social and structural levels.

### Individual and social facilitators

We found that individual factors related to ART adherence included three themes: knowing how ART controls the virus, the inclusion of ART in scheduled routines, and sound psychological health, which seemed to be influenced by social support received from two social groups: clinic peers, and family.

Chäti, Chiko, Jichi, Icho, Belisi, Metiko, Mirono, and Jebi mentioned they knew how ART controls the infection but does not cure HIV. Belisi and Jichi similarly stated, “I know there is no cure; my medicine is (used) to control the virus." In addition, some participants indicated adherence was easier when including ART as part of their daily routine. For example, Mirono, Chäti, and Metiko, and Belisi indicated it was easy for them to remember to take ART because they take their dose every night before bed at the same time.

Good psychological health was found to be directly influenced by social support the participant received from clinic peers and family. For example, Mirono, Cheyei said they found social support and inspiration to maintain adherence and retention in care while talking to other patients at the clinic. A social woker organized a temporary peer support group at the southern ART clinic in previous years. Cheko, Jichi, Chilo reported that the group helped them feel supported, which, they said, translated into better retention and adherence.

Patient social support also came from family members. For example, Chiko, Chichiko, Jebi, Nechi, Mirono, Choi, Kötä, Chöli, and Itoli indicated that family support was crucial for ART adherence. Often, one family member knew the patient was living with HIV and supported them emotionally by checking in. Consequently, the individual wanted to continue with ART. For Mirono, Kötä, Chiko and Jebi, family support also impacted retention in care, as family members often gave the patient financial assistance for monthly travel to the ART clinic.

### Structural facilitators

Two main structural facilitators were elicited that affected ART adherence and retention in HIV care: ART clinic expansion and nutritional supplements. The Ministry of Health provided structural support. The first ART clinic opened in 2010 in the Southern regions Nedrini and Kädriri; in 2017, a second clinic was opened in the Northern region of Ñö Kribo. The new clinics have aided in decreasing travel costs and distance for many patients thereby increasing retention in care. Participants also indicated that both clinics supply nutrient enhanced porridge (*crema*) to all patients at ART retrieval. Some patients (Pancho, Jichi, Belisi, and Itoli) notably said that they would not have eaten food at night when they take their dose if it were not for the *crema*. This nutritional supplementation was found to increase ART adherence.

### Individual and social barriers

We found individual barriers to include lifestyle choices and psychological health. An individual´s lifestyle choices, such as drinking and partying, were found to increase the chances of unprotected sex (Pancho) and decrease regular adherence (Chöli, Cheyei, Pancho). Chöli said, “When there’s a party, and I drink…sometimes I forget to take my medicine.” Despite some mentioning these lifestyle choices, most participants indicated they did not consume alcohol or party.

We found evidence that individual factors, such as the patient’s psychological health, influenced feelings of depression and loss of motivation for adherence. In addition, psychological health was founded in discrimination from various aspects of the participant’s social circle: family, friends, community members, and religious groups, which in turn impacted the patient’s ART retention and adherence.

Family-level discrimination was felt by Chiko, Choi, Cheko, Nichi, Pancho, Jichi, and Itoli. These participants indicated a link between family discrimination and feelings of isolation and depression, which in turn led to apathy towards ART adherence. Chiko, Choi, Cheko, Nichi, Pancho, and Jichi told of being segregated from their family during day-to-day routines, including cooking, eating with family, and sharing sleeping spaces. Cheko explained the effects of family-level discrimination on ART adherence: “Your family is your base, if your family rejects you, what kind of strength do you have to live?… then you don’t take your medicine and you get an infection and die.”

Other patients feared community status disclosure and community-level discrimination, which affected mainly retention in HIV care. Mechi, Cheyei and Mirono said they worried community members would raise questions about their whereabouts when they left their community every month on the same date. Mirono, Jebi, Cheyei, Mechi, Joti, Kötä, Metiko, Cheko, Jichi, and Choi said they had felt discrimination from friends and community members. Many indicated this led them to isolate and not seek further social interaction. Participants, Joti, Icho, Belisi, Jichi, and Choi reported that despite community-level discrimination, the emotional support they received from their family counteracted feelings of isolation; adherence and retention in care were not affected.

### Structural barriers

Structural barriers to ART adherence and retention in care included four general themes: friction between Western/Traditional systems (which affected both adherence and retention), food security (which affected both adherence and retention), medication shortages (which affected adherence), and difficulties to access the ART clinic due to distance and cost (which affected retention in care).

Many participants felt they had to choose between Western and Traditional medicine for three reasons: 1) both Traditional and Western providers told them not to use pluralistic treatments simultaneously; for example, Poncho and Kötä indicated that Traditional providers told them not to mix treatments because ART would lessen the effects of *botanica* (herbal medicine); Chichiko, Itoli, Chilo, Jebi, Belisi, and Cheko said Western doctors feared unknown side effects from mixing treatments; 2) due to the high price of some Traditional providers, Joti, Cheyei, and Jichi were left without traveling money to go to the ART clinic; for example, Joti said, “My family wanted me to take *botanica* and they sold a cow to pay for it, but then we didn’t have more money for me to travel to the (ART) clinic.”; 3) Traditional providers are believed to be able to offer a cure for HIV, as reported by Jebi, Nichi, Chilo, Pancho and Chöli. Additionally, Chilo and Chöli even indicated they had interrupted treatment at least once because they believed their Traditional provider could cure them. In addition, participants reported that some Traditional providers told them they could be cured by believing that God and *botanica* would heal them.

A second structural barrier for several patients was food security. Icho, Pancho, Chichiko, Choi, and Kötä said they were told to take their pills with food, but often they did not eat in the evenings. Chichiko notably said, “They told me to eat before I take my pills, but my family doesn’t eat at night… So, I didn’t know if I should take my medicine.” To lower ART side effects, patients are advised by Western clinicians to take medicine at night with food. However, traditionally and perhaps due to poverty, many individuals in CNB only eat one meal a day.

All participants mentioned having their treatment interrupted at least once due to ART supply shortage at the clinic. Cheko said, “In the 16 years I have been on treatment, the clinic has run out of pills for a while at least once a year. Sometimes I don’t get my pills for a month or three, sometimes they say to come the following week to pick them up, but then I don’t have money to travel again… when the pills come in and I can travel, I start taking them again.”

The last structural barrier we found is access to the ART clinic due to the distance and the cost of travel. Kötä explained that he walks 8 hours to get to a road where there is a bus down to the clinic. Metiko and Chöli indicated they could not travel to and from the clinic in one day because of the long distance from their home. Therefore, further costs are incurred with finding where to stay, or more disconcertingly, they may sleep outside the clinic (like Pancho did). Chilo, Chäti and Jichi said they relied on family to give them money to travel. However, at times, family members cannot provide for the transport costs either; therefore, the patient may miss picking up the treatment for that month or altogether drop out of care. Chäti said, “Sometimes my father tells me it’s too expensive (to keep me in care), so I want to stop taking pills to not be a burden."

## Discussion

The CNB is a relatively isolated and poor region of Panama, with deficient access to ART and HIV clinics, high ART and HIV care dropout rates, resulting in poor virological control and high HIV-related mortality. However, to our knowledge, there has not been any prior research to elicit the factors that influence adherence to ART in the CNB. Study participants reported several individual/social facilitators and individual/social and structural barriers to ART adherence and retention. Our study found that facilitators and barriers in care are based within the interaction of individual/social/structural levels, as described in Bronfenbrenner’s mesosystem [18]. Interestingly, we did not find differences in gender regarding ART adherence or retention in the responses. In other low- and middle-income settings, men have higher rates of AIDS-related mortality, due at least partially to maintaining traditional masculinity [19]. Some of the factors related to traditional masculinity include the perspective that HIV and clinical care affect men´s traditional roles such as physical strength, risk-taking and the perspective that medical clinics are feminine spaces [20]. In addition, national data do show a stark difference between mortality rates in the CNB compared to national rates. Future research should be undertaken to understand the discrepancy in these geographical regions.

Individual-level factors were based on psychological health were mainly related to social acceptance/discrimination. Our participants indicated that retention in care was especially challenging as participants feared disclosure of their serostatus and because of family-level discrimination. The association of good mental health with social support at the foundation has been described as the backbone of both ART adherence and retention in care [21] and has impacted interpersonal, psychological and mental health, especially anxiety and depression [22, 23].

We found that not all social exclusion affects the participant’s adherence and retention the same way. For example, family-level discrimination was detrimental to psychological well-being, and to retention in care, and consequently ART adherence. However, although community-level discrimination affected participants, simultaneous family support seemed to counteract adverse adherence/retention outcomes. Studies in Thailand and the United States have reported the importance of family support in ART retention and adherence, especially for young people [24, 25].

Structurally, we have documented regular ART shortages in the clinics, which impact on ART adherence. Additionally, patients use two parallel health systems (Western and Traditional) that often do not complement each other, and may instead often compete for patients. Ngäbe and Buglé peoples often choose the geographically and culturally closer provider, even if Traditional provider fees are high. Western clinicians were sought when the Traditional medicine fails viral control. The uncooperative interaction between the two health systems could be detrimental to some participants´ ART adherence and retention in the Western HIV care system. Other countries have seen similar results. For example, the use of traditional healers in Senegal has been correlated with more advanced HIV/AIDS disease and increased mortality [26]. Medical pluralism, the use of multiple medical systems such as Traditional and Western systems simultaneously or interchangeably, is especially popular in chronic conditions such as HIV [27]. Use of Traditional medicine, which is widespread across the CNB [28], may be favored by patients because of the focus on holistic and culturally congruent care [29]. Despite existing plurality, patients reported that both systems asked them not to use simultaneous treatments. A multicounty study in Eastern and Southern Africa, found medical pluralism significantly created delays and interruptions in HIV care throughout the cascade; authors cite the need to minimize consequences of system pluralism through culturally sensitive interventions [30].

### Implications for policy and practice

Families are often overlooked in ART care interventions. However, family systems are central to social functioning in the CNB [31]. We found that family support was vital for adherence and retention. Interventions with families have been found to support positive HIV outcomes, where parents or other family members at home served as adherence guides and sources of HIV knowledge [24, 25]. This at-home ´buddy system´ was associated with a decrease in HIV viral load and increase other health outcomes such as dental appointments and immunizations [25]. Broader social-network and community-level approaches could increase societal knowledge and support while decreasing stigma, as shown in Kenya and Zimbabwe [32, 33]. For example, these studies have found that that the inclusion of community and members of social networks in the management of HIV through group training has significant effects on viral suppression and reduction of treatment interruptions in adults and children [32, 33]. These interventions could be part of existing HIV care to not to lose patient continuity. In CNB, some participants mentioned the success of the temporary, professionally guided, peer-support group; this group should be revived and expanded. In Zimbabwe, the Friendship Bench, a low-cost therapy intervention run by lay healthcare workers, was found to be effective mental-health support for PLHIV [34]. In the CNB, it may be advantageous to try a similar program incorporating patients and family members.

A major structural barrier related to ART access was influenced by cost and distance to travel to the clinic. This barrier was, without a doubt, exasperated by the COVID-19 pandemic in 2020 and 2021, where the *Covidization* of healthcare significantly influenced HIV care [35]. Prior to the pandemic, we found that patients were expected to retrieve ART monthly and participate in biannual CD4 count and/or HIV plasma viral load check-ups during our data collection. Although ART is provided for free in Panama [8], monthly clinic trips may be too much of a financial burden to continue treatment, as found in a worldwide review [36]. In June 2020, after months of clinic closures and decreased hours due to the COVID pandemic, there was a felt need for multi-month ART dispensing in the CNB. When there is enough ART in stock, patients may receive up to 3 months dispensed at a time. Other models of differentiated delivery of ART include fast-track drug refills for up to 6 months. In the CNB, 6-month refills and decentralized services to smaller health centers across the country could decrease the time and money spent on pill pick-up. For example, home-based visits in China, Uganda, and Rwanda have increased ART adherence and viral suppression [37–40]. Task shifting to dispense ART through non-specialized health workers could also decrease the need for travel, thereby increasing adherence and clinical outcomes, as shown in several studies [40–44]. However, newer research has found that community-based drug delivery could be costly to the clinics and could force clients to identify as living with HIV, a stigmatising albeit unvoluntary disclosure [45].

Food insecurity at the household level has been shown in a worldwide review to be a limiting factor to ART adherence, due to more significant side effects of ART when taken without food, and competing economical use demands when choosing between clinic travel or food [46]. Despite existing supplemental nutrition in CNB, some PLHIV still experienced food scarcity. Therefore, the provision of food items should be considered in some ART programs. A study in Honduras found an increase in adherence when a monthly food basket was provided at the point of ART retrieval [40, 47].

In CNB, the relationship between Western and Traditional health systems was exclusionary. However, these systems could coexist for successful patient care, as is found in other low- and middle-income countries, for example in South Africa [48]. Successful collaborations could decrease potential morbidity and mortality related to potentially unsafe practices and low ART adherence and poor HIV care retention rates [48–50]. Further complicating these interactions, is the promise of cure by some Traditional providers in CNB. Similar findings have emerged in Uganda [51] and Tanzania [52]. Due to the overwhelming use of Traditional medicine throughout the CNB, Western medicine could benefit from the trust and cultural closeness by systematically including Traditional providers to help deliver care. Interventions could incorporate Traditional providers for ART promotion and patient home visits. Traditional healers also play an important role in Mozambique; the Western system has engaged Traditional healers as community health workers as ART adherence partners, especially in areas where Western care is lacking [53]. However, despite Western and Traditional providers recognizing the importance of collaboration, in Zambia, institution-based, one-way teaching methods have been unsuccessful in promoting collaboration between the two systems [54].

Our study found consistent findings with a diverse group of PLHIV, including adherent/intermittently adherent patients. We also included a nearly identical participant sex ratio to CNB clinic statistics, as 82% of PLHIV in CNB are male [55]. As no gender differences in responses were elicited in this initial study, we believe structural barriers may overshadow gender-based differences related, at least in part, to traditional masculine norms, such as the perspective that HIV and clinical care are feminine spaces [20]. Further research should investigate possible gender differences in AIDS-related mortality rates. Despite these strengths, this study had some key limitations. Firstly, the interviewers, AG and ES, are ethnically white women, which may have decreased accurate reporting among Indigenous participants. However, HIV status in CNB is generally seen as a sensitive and confidential topic; the fact that the interviewers were markedly from outside the CNB may have increased openness as the risk of divulging serostatus among community members decreased. Secondly, semi-structured interviews were undertaken in Spanish, which may have limited responses from participants, although Spanish is used in public institutions, especially schools and health centers, and over 70% of CNB individuals have attended basic Western education [2, 3]. Lastly, patient representativeness may be biased since ART clinicians were directly involved in selecting participants, and non-adherent patients who had dropped out entirely of HIV care could not be contacted, for ethical and practical reasons. However, our study participants represent a range of adherence levels from previously non adherent to intermittently adherent and fully adherent, which are commonly encountered in ART clinic settings.

## Conclusions

This qualitative study among PLHIV focused on a relatively isolated group of Indigenous peoples who live in a remote rural region of Western Panama. Our findings, especially on social and material levels, are similar to those in other low- and middle-income settings, especially to those found in some sub-Saharan African studies. These similarities indicate the universality of some barriers to ART adherence and retention in care, especially in regions where traditional healers play an important role in care. However, this is the first study to examen this among this topic among Indigenous people of the CNB, adding to the literature on Indigenous health and HIV care. In the CNB, we found that participants’ experience in ART adherence and retention in care was strongly influenced by interrelated individual, social, and structural factors. These factors included individual psychological health and family and community social support or discrimination. Notably, PLHIV in the CNB have difficulties deciding between Traditional and Western health systems offer because of the travel distance and cultural closeness. Additionally, poverty and food insecurity made adherence difficult for many, while others were affected by recurrent ART shortages. Finally, as Panama’s HIV epidemic continues, we propose several interventions which have been evaluated in other LMICs and that could be undertaken in the CNB to address the social and structural-level barriers to ART adherence and retention in HIV care.
